# Emergent Management of Growing Teratoma Syndrome: A Case Presentation

**DOI:** 10.7759/cureus.59335

**Published:** 2024-04-30

**Authors:** Devyansh Nimodia, Pratap Parihar, Vadlamudi Nagendra, Harshith Gowda, Prasad Desale

**Affiliations:** 1 Radiodiagnosis, Jawaharlal Nehru Medical College, Datta Meghe Institute of Higher Education and Research, Wardha, IND

**Keywords:** chemotherapy, surgical excision, pulmonary metastasis, retroperitoneal mass, non-seminomatous germ cell tumor, growing teratoma syndrome

## Abstract

Growing teratoma syndrome (GTS) represents a rare yet significant complication following treatment for non-seminomatous germ cell tumors (NSGCT), characterized by the growth of mature teratoma elements despite prior chemotherapy. We present the case of a 30-year-old male who, following orchidectomy for NSGCT and subsequent chemotherapy, developed acute abdominal pain and pulmonary metastasis. Despite normal serum tumor markers, imaging revealed a large retroperitoneal mass encasing significant vessels. Surgical excision led to symptom resolution. This case underscores the diagnostic challenges GTS poses, the importance of imaging in diagnosis, and the efficacy of prompt surgical intervention in achieving favorable outcomes.

## Introduction

Non-seminomatous germ cell tumors (NSGCT) constitute a diverse group of malignancies arising from primordial germ cells, predominantly affecting young males between 15 and 35 [1]. Management typically involves a multimodal approach, including surgical resection and chemotherapy, with the combination regimen of bleomycin, etoposide, and platinum (BEP) being the standard of care [2]. Despite advances in treatment, a subset of NSGCT patients experience relapse or develop complications post-treatment, posing significant challenges in their management. One such rare but clinically relevant complication is growing teratoma syndrome (GTS), first described by Logothetis et al. in 1982 [3]. GTS is characterized by the growth of mature teratoma elements within the residual tumor mass following chemotherapy, despite the normalization of serum tumor markers [4].

The pathogenesis of GTS remains poorly understood, with hypotheses suggesting incomplete eradication of teratoma cells by chemotherapy or dedifferentiation of malignant cells into more resistant phenotypes [5]. Diagnosis of GTS relies on clinical presentation, imaging studies, and histopathological examination of the resected tumor specimen [6]. Serum tumor markers, including alpha-fetoprotein (AFP) and beta subunit of human chorionic gonadotropin (β-hCG), may be within normal limits, further complicating diagnosis [7]. Management of GTS involves a prompt surgical intervention to achieve complete excision of the residual tumor mass. This approach alleviates symptoms and minimizes the risk of malignant transformation and potential metastasis [8]. Long-term prognosis following surgical resection of GTS is generally favorable, with reported recurrence rates ranging from 10% to 30% [9]. Given the rarity of GTS and its potential for life-threatening complications, it is essential for clinicians to maintain a high index of suspicion in NSGCT patients presenting with recurrent or progressive symptoms post-treatment. This case report aims to underscore the clinical manifestations, diagnostic challenges, and therapeutic considerations associated with GTS, emphasizing the significance of timely surgical intervention in optimizing patient outcomes.

## Case presentation

A 30-year-old male patient presented to the medical oncology outpatient department at our rural hospital with complaints of acute generalized abdominal pain radiating to his back. He also reported mild distention of the abdomen and dull, aching pain that gradually developed over time. The patient had a previous history of right testicular pain, for which he sought medical attention at a local government hospital. He underwent a right orchidectomy, and the histopathology report confirmed an advanced NSGCT, the sample of which we were unable to obtain from the patient. Subsequently, he received four cycles of chemotherapy, comprising three cycles of BEP and one cycle of etoposide and platinum (EP), with the last cycle being completed one month ago.

The need for further follow-up and treatment prompted the patient's referral to our hospital. He denied any history of vomiting, bowel complaints, fever, or trauma and reported no comorbid conditions. His blood count and hemoglobin level were within the normal range. However, his liver function test indicated elevated levels of AST at 323. Although the serum markers typically associated with GTS (such as ALF and β-hCG) were within normal limits, the lactate dehydrogenase (LDH) level was increased. It is worth noting that the serum markers for GTS often remain within the normal range. All other parameters were found to be within normal limits.

**Figure 1:**
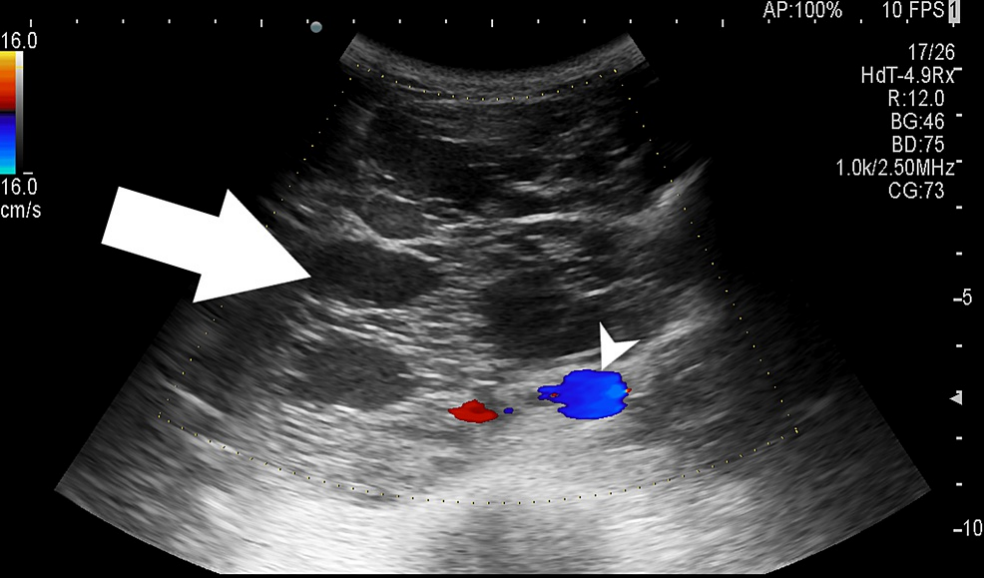
Ultrasound gray-scale image of the abdomen showing a large ill-defined multicystic mass in the retroperitoneum (arrow) with no internal vascularity on color flow. Arrowhead showing abdominal aorta being encased

Chest X-ray revealed multiple nodular opacities in bilateral lung fields suggestive of pulmonary metastasis. Ultrasound of the abdomen and pelvis revealed a large retroperitoneal ill-defined multicystic mass with echogenic walls and no flow on color Doppler, causing lifting and encasement of the aorta and inferior vena cava. No intralesional calcifications were noted (Figure 1).

**Figure 2:**
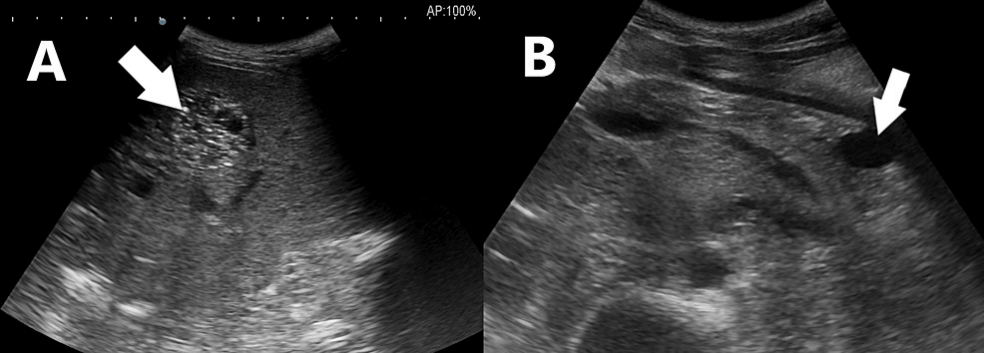
The gray-scale section of the liver in A shows a well-defined hyperechoic mass (arrow) and B shows a well-defined cystic lesion in the trail of the pancreas (arrow)

Multiple variable-sized hyperdense lesions were observed in the liver, along with a cystic lesion in the distal body of the pancreas (Figure 2).

**Figure 3:**
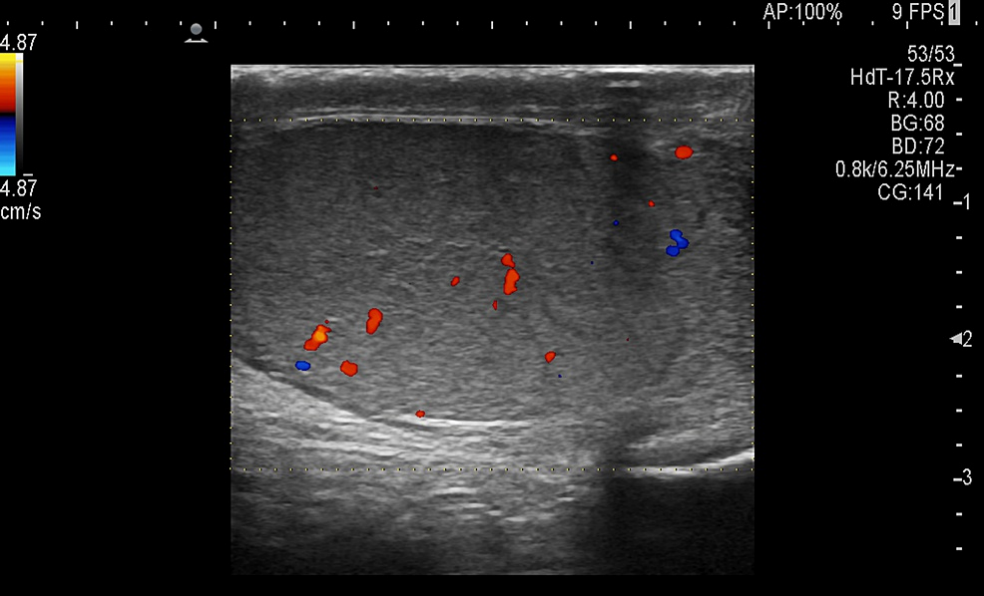
Ultrasound image showing normal appearance of the left testis with normal vascularity on color flow

The right testis was not visualized, indicating its post-operative status, while the left testis appeared normal in size, shape, echotexture, and vascularity (Figure 3).

**Figure 4:**
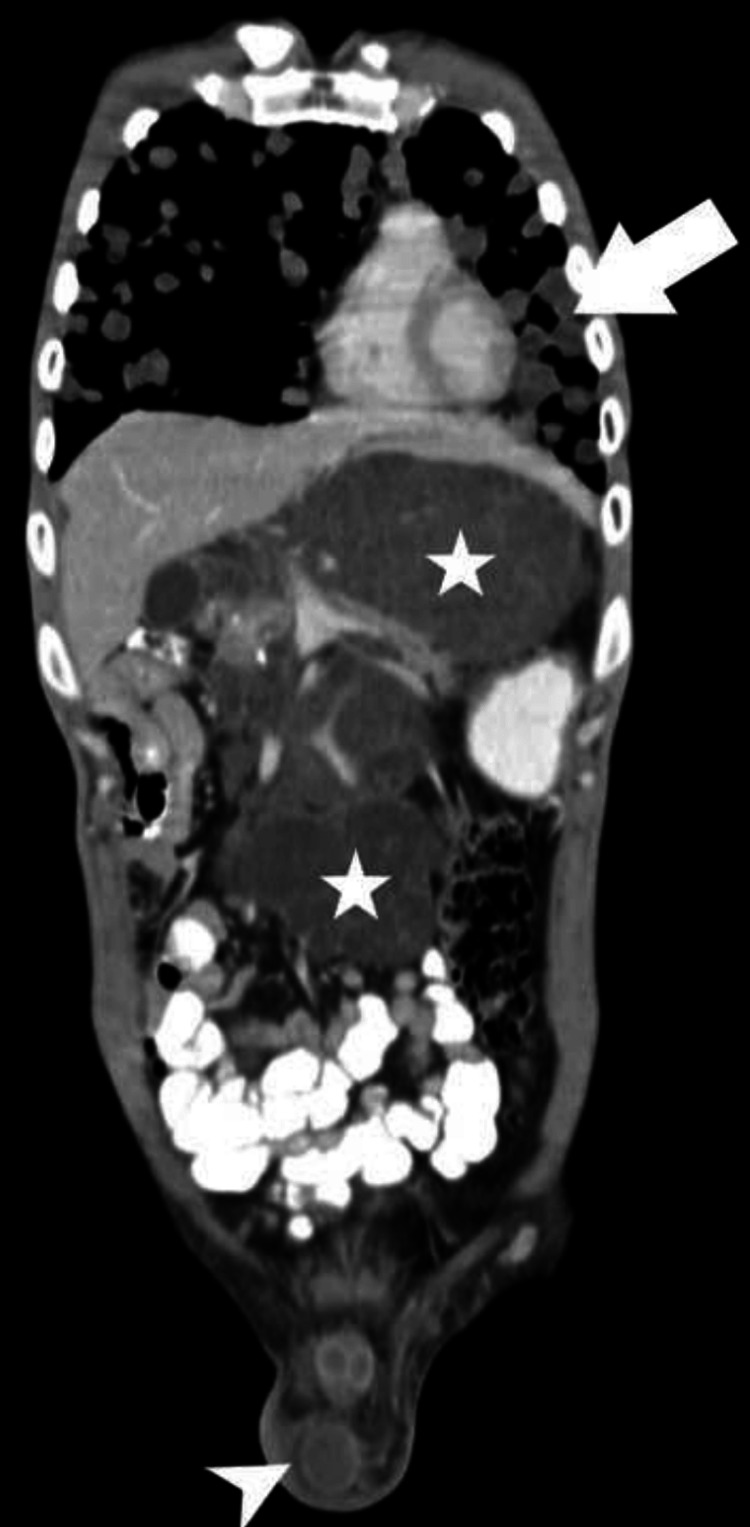
CT mediastinal window showing multiple variable-sized cannon ball metastasis (arrow) in bilateral lung parenchyma. Contrast CT abdomen venous phase showing enhancing lesion (star) in segment VII of liver measuring 5.2 x 4.3 cm and ill-defined retroperitoneal mass (arrowhead) CT: computed tomography

**Figure 5:**
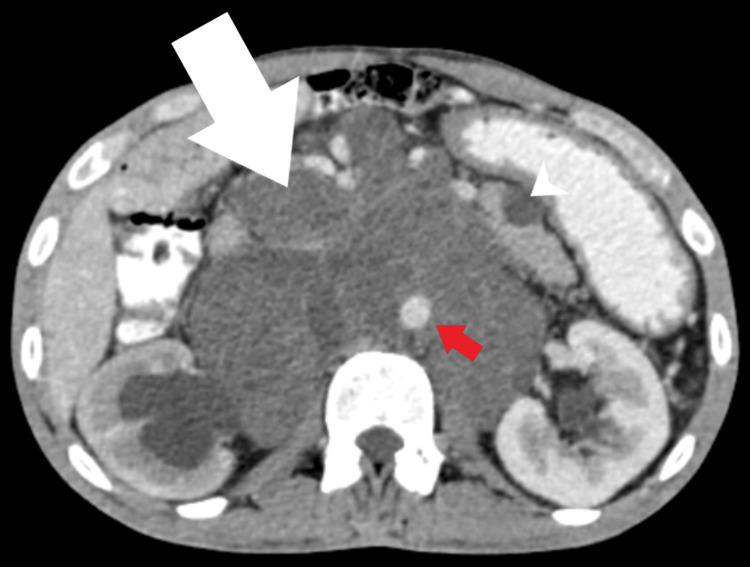
Large peripherally enhancing ill-defined retroperitoneal cystic mass (arrow) causing encasement and lifting abdominal aorta (red arrow). The lesion is compressing the right mid ureter causing right obstructive hydronephrosis. Enhancing cystic lesion seen in the tail of pancreas measuring 1.6 x 1.5 cm (arrowhead)

Contrast-enhanced computed tomography (CT) scans of the thorax and abdomen further confirmed the findings, suggesting pulmonary metastasis (Figure 4) and a large peripherally enhancing retroperitoneal lesion, respectively, encasing major blood vessels and causing ureteral compression and dilatation of the pelvicalyceal system (Figure 5).

**Figure 6:**
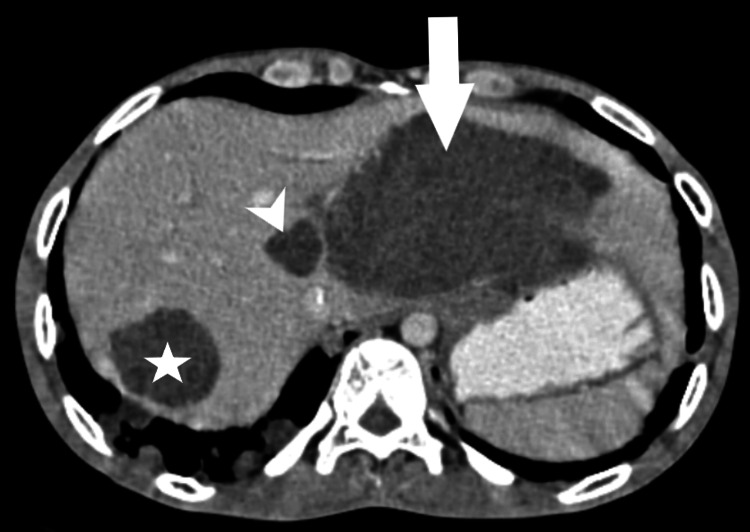
Contrast CT y axial abdomen venous phase showing multiple enhancing lesions in the liver (star and arrowhead), largest in segment VII (asterisk) CT: computed tomography

One week post-admission, the patient underwent a surgical fitness assessment, and the subsequent day, underwent complete excision of the tumor. A post-operative CT scan indicated complete resolution of the retroperitoneal tumor (Figure 6). The patient was discharged with instructions to rest and attend bi-weekly follow-up appointments.

## Discussion

GTS is a rare but clinically significant complication that can arise following treatment for NSGCT. It is characterized by the growth of mature teratoma elements within previously treated germ cell tumor sites despite previous chemotherapy or radiotherapy. Our case report illustrates the diagnostic and therapeutic challenges posed by GTS and emphasizes the importance of timely recognition and intervention. GTS was first described by Logothetis et al. in 1982 and has since been recognized as a distinct clinical entity [10]. It typically occurs in patients who have undergone chemotherapy for NSGCT, as illustrated in our case, where the patient had received multiple cycles of chemotherapy, including BEP and EP regimens. Despite an initial response to chemotherapy, GTS can develop due to the persistence of teratoma elements resistant to chemotherapy [11].

Diagnosing GTS can be challenging, as serum tumor markers commonly used to monitor NSGCT, such as AFP and β-hCG, may remain within normal limits [12]. In our case, the patient's serum tumor markers were within normal limits, highlighting the limitations of relying solely on these markers for detecting GTS. Imaging studies, including ultrasound and CT scans, are crucial in diagnosing GTS. In our case, CT scans revealed a large retroperitoneal mass encasing major vessels and causing ureteral compression, consistent with GTS. Surgical intervention is the mainstay of treatment for GTS, as illustrated in our case, where the patient underwent complete excision of the retroperitoneal mass, resulting in symptom resolution. Surgical resection aims to achieve complete tumor removal and prevent further growth or dissemination. However, the timing of surgery remains a subject of debate, with some advocating for early intervention to prevent complications such as organ compression or vascular compromise [13]. The prognosis of GTS varies depending on factors such as the extent of disease, response to treatment, and histological characteristics of the tumor. Despite advancements in surgical techniques and adjuvant therapies, the recurrence rate of GTS remains high, emphasizing the need for close long-term follow-up and surveillance [14].

## Conclusions

In conclusion, our case report highlights the importance of considering GTS as a potential complication following treatment for NSGCT. Despite normal serum tumor markers, imaging studies played a crucial role in diagnosing GTS, revealing a retroperitoneal mass encasing significant vessels. Prompt surgical intervention led to successful tumor excision and resolution of symptoms, underscoring the significance of timely recognition and intervention in managing GTS. However, the recurrence rate of GTS remains high, emphasizing the need for long-term follow-up and surveillance in patients with NSGCT. Further research is warranted to better understand GTS's pathogenesis and improve treatment outcomes for affected individuals.
